# Aaptamine – a dual acetyl – and butyrylcholinesterase inhibitor as potential anti-Alzheimer’s disease agent

**DOI:** 10.1080/13880209.2022.2102657

**Published:** 2022-08-14

**Authors:** Shuang Miao, Qianqian He, Chen Li, Yan Wu, Mengshan Liu, Yongshou Chen, Shizhou Qi, Kaikai Gong

**Affiliations:** a Medical Research Center, Binzhou Medical University Hospital, Binzhou, PR China; b Department of Pharmacy, Binzhou Medical University Hospital, Binzhou, PR China; c Key Laboratory for Green Chemical Process of Ministry of Education, School of Chemical Engineering and Pharmacy, Wuhan Institute of Technology, Wuhan, PR China

**Keywords:** Aaptamine, Alzheimer’s disease, acetylcholinesterase, butyrylcholinesterase, surface plasmon resonance, molecular docking, zebrafish, dyskinesia rehabilitation

## Abstract

**Context:**

Alzheimer’s disease (AD) is a neurodegenerative disorder that affects millions of people worldwide. Acetylcholinesterase (AChE) and butyrylcholinesterase (BuChE) are promising therapeutic targets for AD.

**Objective:**

To evaluate the inhibitory effects of aaptamine on two cholinesterases and investigate the *in vivo* therapeutic effect on AD in a zebrafish model.

**Materials and methods:**

Aaptamine was isolated from the sponge *Aaptos suberitoides* Brøndsted (Suberitidae). Enzyme inhibition, kinetic analysis, surface plasmon resonance (SPR) and molecular docking assays were used to determine its inhibitory effect on AChE and BuChE *in vitro*. Zebrafish were divided into six groups: control, model, 8 μM donepezil, 5 , 10  and 20 μM aaptamine. After three days of drug treatment, the behaviour assay was performed.

**Results:**

The IC_50_ values of aaptamine towards AChE and BuChE were 16.0 and 4.6 μM. And aaptamine directly inhibited the two cholinesterases in the mixed inhibition type, with *K_i_* values of 6.96 ± 0.04 and 6.35 ± 0.02 μM, with *K_d_* values of 87.6 and 10.7 μM. Besides, aaptamine interacts with the crucial anionic sites of AChE and BuChE. *In vivo* studies indicated that the dyskinesia recovery rates of 5 , 10  and 20 μM aaptamine group were 34.8, 58.8 and 60.0%, respectively, and that of donepezil was 63.7%.

**Discussion and conclusions:**

Aaptamine showed great potential to exert its anti-AD effects by directly inhibiting the activities of AChE and BuChE. Therefore, this study identified a novel medicinal application of aaptamine and provided a new structural scaffold for the development of anti-AD drugs.

## Introduction

Alzheimer’s disease (AD) is an age-related neurodegenerative disorder characterized by memory loss and behavioural disturbances; it has a serious impact on human health and life. As the population ages, the number of patients is expected to reach 130 million worldwide by 2050 (Lane et al. 2018; Bowroju et al. 2020). According to previous research, the main hallmarks of AD include the accumulation of β-amyloid in senile plaques, hyperphosphorylated neurofibrillary tangles of tau protein, and loss of cholinergic activity in certain parts of the brain (Demir Özkay et al. 2016). Cholinergic deficits resulting in the loss of brain-cell communication are the most popular hypothesis (Weller and Budson 2018). Currently, four FDA-approved drugs, namely donepezil, rivastigmine, galantamine and tacrine are cholinesterase (AChE) inhibitors; tacrine was removed from the market in 1998 due to hepatotoxicity (Dhillon 2021). They are commonly used to treat mild-to-moderate AD and can only slow down neurodegeneration in patients with AD to some extent (Gomes et al. 2019; Saxena and Dubey 2019). Thus, the exploration of novel drug candidates with consistent therapeutic potential and fewer side effects is urgently needed.

The maintenance of acetylcholine (ACh) levels by inhibiting cholinesterase activity has been proven to be a successful way to relieve some cognitive and behavioural symptoms of AD (Ferreira-Vieira et al. 2016). In addition to the widely known AChE, butyrylcholinesterase (BuChE) has also been confirmed to metabolize ACh. In the advanced stages of AD, the enzymatic activity of AChE is decreased, whereas the activity of BuChE is stable or even increased. Furthermore, the ratio of BuChE to AChE has also increased, ranging from 0.2 to 11.0 in certain areas of the brain. This indicated that BuChE may act as a compensatory mechanism for ACh metabolism (Andrisano et al. 2018; Košak et al. 2018). Furthermore, *in vivo* experiments revealed that AChE and BuChE also play an important role in the production and aggregation of Aβ peptides in the brains of AD patients (Sakayanathan et al. 2019). Moreover, increasing evidence supports the idea that dual AChE/BuChE inhibition can maximize the therapeutic efficacy in AD (Kandiah et al. 2017; Yang et al. 2019). Therefore, discovering novel AChE/BuChE dual-targeted inhibitors can be a more effective approach for the treatment of AD.

To discover new classes of AChE/BuChE dual-targeted inhibitors from natural products, we performed a chemical investigation of the marine sponge *Aaptos suberitoides* Brøndsted (Suberitidae) and obtained a series of aaptamine alkaloids; the main chemical constituent, aaptamine, exhibited the most potent inhibitory activities towards both AChE and BuChE. We then performed kinetic assays and molecular docking to predict the inhibition types of aaptamine against AChE and BuChE. Additionally, a surface plasmon resonance (SPR) assay was performed to measure the affinity of aaptamine for the two cholinesterases.

Zebrafish displays a high degree of similarity with the neuroanatomical and neurochemical pathways in humans. Thus, they have become an attractive and valuable model for drug screening because of certain advantages, including their small size, short generation time, rapid development and large numbers of offspring (Santana et al. 2012; de Abreu et al. 2020). Published articles have shown that the aluminium chloride (AlCl_3_)-induced zebrafish AD model can be used for screening potential anti-AD lead compounds (Caramillo and Echevarria 2017; Saleem and Kannan 2018; Pitchai et al. 2019). In this study, an AlCl_3_-induced zebrafish AD model was established as the *in vivo* animal model to evaluate the therapeutic effects of aaptamine.

## Materials and methods

### Reagents and chemicals

Aaptamine with a purity of more than 98%, was isolated from marine sponge *A. suberitoides.* AChE (200 U/g from electric eel) and 5,5′-dithio*bis*(2-nitrobenzoic acid) (DNTB) were purchased from Macklin Inc. (Shanghai, China). BuChE (10 U/mg from horse serum), acetylthiocholine iodide (ATChI) and butyrylthiocholine iodide (BTChI) were purchased from Shanghai Yuanye Bio-Technology (Shanghai, China). Donepezil was obtained from the Sigma-Aldrich (Darmstadt, Germany).

### Extraction and isolation of aaptamine

**Figure 1.**
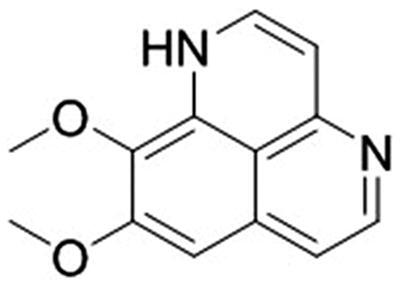
Chemical structure of aaptamine.

The marine sponge *A. suberitoides* was collected from the South China Sea in October 2012. The specimen was identified by Dr. Nicole J. de Voogd (National Museum of Natural History, Leiden, Netherlands). The voucher specimen (NO. XS-2012-30) was deposited at State Key Laboratory of Marine Drugs, Ocean University of China, Qingdao, Shandong, China. The isolation of aaptamine was described in our previous work (Gong et al. 2020). Briefly, the MeOH extract of *A. suberitoides* was subjected to column chromatography (CC) on silica gel using petroleum ether/acetone (from 100:1 to 1:1, *v/v*) and dichloromethane/methanol (from 20:1 to 0:1, *v/v*) as eluent to obtain nine fractions (Fr.1–Fr.9). Fr.6 was separated by silica gel CC and Sephadex LH-20 to yield Fr.6.6.1–Fr.6.6.3. Fr.6.6.2 was further purified by semi-preparative RP-HPLC (C18, MeOH/H_2_O, 40:60, *v/v*, 1.5 mL/min) to yield compound aaptamine (62.5 mg) (Figure 1). The spectroscopic data was consistent with data previously reported for aaptamine.

Aaptamine: C_13_H_12_N_2_O_2_, yellow oil, ^1^H NMR (500 MHz, DMSO-*d*_6_) *δ*_H_: 12.82 (1H, s, NH-1), 12.30 (1H, s, NH-4), 7.85 (1H, d, *J* = 6.8 Hz, H-2), 7.39 (1H, d, *J* = 6.6 Hz, H-5), 7.10 (1H, s, H-7), 6.86 (1H, d, *J* = 7.0 Hz, H-6), 6.39 (1H, d, *J* = 6.8 Hz, H-3), 3.97 (3H, s, OMe-8), 3.81 (3H, s, OMe-9); ^13^C NMR (125 MHz, DMSO-*d*_6_); *δ*_C_: 156.9 (C-8), 149.7 (C-3a), 141.9 (C-2), 133.7 (C-9a), 132.6 (C-6a), 131.4 (C-9), 139.9 (C-5), 116.3 (C-9b), 112.6 (C-6), 100.9 (C-7), 98.1 (C-3), 60.3 (OMe-9) and 56.5 (OMe-8).

### *In vitro* AChE and BuChE activity inhibition assay

The lyophilized powder of AChE and BuChE was dissolved in protein stabilizer (0.1 g BSA dissolved in 100 mL PBS) to form the storage concentration of 10 U/mL and then diluted to the required concentrations. The compound aaptamine and the positive control donepezil were dissolved in DMSO (1%) and diluted with PBS (pH = 7.4) to obtain different concentrations. The inhibitory activities of aaptamine and donepezil towards AChE and BuChE were assayed by the spectrophotometric method developed by Ellman et al. with slight modification. In a 96-well microplate, 68 μL of PBS, 20 μL of DTNB (5 mM), 1 μL of AChE or BuChE (4 U/mL) and 1 μL of aaptamine or donepezil were added. After 30 min of incubation at 37 °C, 10 μL of substrate ATChI or BTChI (10 mM) were added and incubated at 37 °C for 30 min. For the control group, all components were added except aaptamine and donepezil. For the blank control group, the assays were carried out with wells containing all components except AChE and BuChE. The samples were measured the absorbance at 412 nm by spectrophotometer (BioTek, Synergy 2, Winooski, VT) and all the wells were deducted the background. The inhibition rate (%) = 100 − (Abs_sample_ – Abs_blank_)/(Abs_control_ – Abs_blank_) × 100. The minimum inhibitory concentration to reduce the enzyme activity by 50% (IC_50_) is calculated using the GraphPad Prism version 7.0 software (La Jolla, CA), by a non-linear regression analysis. All experiments were performed in triplicate.

### Docking procedures

Molecular docking was implemented using the surflex-docking package of Sybyl-X 2.1 (Tripos International, St. Louis, MO). The 3D structure of aaptamine was optimized to establish the lowest energy state and saved as mol.2 file format by ChemBio 3D version 17.1.0 software (Massachusetts , USA). Then, the stable 3D structure of ligand was automatically generated by the GeomX docking mode of the sybyl software. Crystal structures of recombinant human AChE in complex with Donepezil (4EY7) and BuChE with *N*-[1-(2,3-dihydro-1H-inden-2-yl)piperidin-3-yl)methyl]-*N*-(2-(dimethylamino)ethyl)-2-naphthamide (4TPK) were obtained from the Protein databank (Cheung et al. 2012; Brus et al. 2014; Sakayanathan et al. 2019). Before docking, the cholinesterases were prepared by removing water, extracting the ligand and addition of hydrogen. Then, the ‘protomol’ was generated using the ligand-based mode, and an appropriate binding pocket was formed. The geomx mode was used for flexidock, and the docking process of aaptamine with one cholinesterase takes about 30 min. The docking used in this topic is local docking. The selected docking region is the spatial position of 5 Å around the protein protoligand. The molecular simulation diagram is drawn by PyMOL software (Seeliger and de Groot 2010).

### Kinetic study for the inhibition of aaptamine on cholinesterases

To clarify the mechanism of action of aaptamine, reciprocal plots of 1/[velocity] *vs.* 1/[substrate] (Lineweaver-Burk plots) were constructed at different concentrations of the substrate ATChI or BTChI (0, 1.25, 2.5, 5, 10 and 25 mM) and aaptamine (0, 5, 20 and 40 μM). Meanwhile, the parallel experiment was also carried out without aaptamine. The remaining procedure was the same as that of described in cholinesterases inhibition assay. The data were plotted on a Lineweaver–Burk diagram using Microsoft Excel 2016 to reveal the mechanism of inhibition. The steady-state inhibition constant *K_i_* (intercept on the x-axis) was determined by the secondary plot of slope *vs.* the concentrations of inhibitor.

### Surface plasmon resonance (SPR) assay

The experiments were carried out using Biacore T100 with the Sensor Chip CM5. The chip surface was activated following standard NHS + EDC with Biacore PBS-EP (0.02 M Phosphate buffer with 2.7 mM KCl, 0.137 M NaCl and 0.05% P20) buffer used as the running buffer. 7000 RU of untagged AChE or 6600 RU of untagged BuChE was immobilized per flow cell and 100 μg/mL AChE or 20 μg/mL BuChE was injected. The cholinesterase protein is coupled to the sensor chip CM5 by amine coupling. 1 M (pH 8.5) ethanolamine-HCl was used to seal off the active group. Aaptamine was injected into the protein channel and blank channel at the following doses: 100, 50, 25, 12.5, 6.25, 3.125 and 0.39 μM at a flow rate of 30 μL/min. The protein binding period was set to 1 min, and the dissociation period was set to 2 min. The affinity was obtained by fitting the binding data using Biacore T100 Evaluation Software version 3.0 (Chicago, USA).

### Efficiencies of aaptamine on AlCl_3_-induced zebrafish AD model

Zebrafish AD model has been widely used in rapid screening of anti-AD potential therapeutic agents (Thawkar and Kaur 2021). Hence, wild type zebrafish larvae were purchased from the drug screening platform of the Institute of Biology, Shandong Academy of Sciences. Briefly, the 3–6 dpf zebrafish larvae were randomly placed in a 6-well microplate at a density of 30 zebrafish per well and treated with 100 μM AlCl_3_ to establish the AD model. Zebrafish was divided into six groups: control, model, donepezil (positive group) and low-, medium- and high-, dose treatment. The control group was maintained in the reverse osmosis water with 0.5% DMSO while the model group was treated with 100 μM AlCl_3_ containing 0.5% DMSO. Based on the data revealed in published articles, 8 μM of donepezil performed well in AlCl_3_-induced zebrafish AD model. Thus, in this experiment, the donepezil group was treated with both 100 μM AlCl_3_ and 8 μM donepezil. In addition, three treatment groups were co-treated with 100 μM AlCl_3_ and different concentrations of aaptamine (5, 10 and 20 μM) containing 0.5% DMSO. After 3 d, the distance and speed change after light stimulus alteration were analysed and recorded for three rhythmic light within 60 min (10 min darkness and 10 min illumination alternately) using viewpoint behaviour analyser (Zebralab V3, ViewPoint Life Sciences Co., Ltd., Lyons, France). The protocol for this study involving animals and their maintenance was approved by the Animal Care and Use Committee of Shandong Academy of Sciences (No.00612).

$$\text{Efficacy on motility }(\%)=[D_{\text{sample group}}-D_{\text{model group}}]/[D_{\text{untreated group}}-D_{\text{model group}}]\times 100.$$

*D* is the movement distance of zebrafish during 60 min.

Efficacy on speed change (%) = [*V*_sample group_ – *V*_model group_]/[*V*_untreated group_ – *V*_model group_] × 100. V is the average velocity (mm/s) of different groups which was obtained by recording the movement distance of every min for every zebrafish during 60 min. All data are expressed as mean ± SEM and analysed by GraphPad Prism version 7.0 software (Graphpad Software Inc., La Jolla, CA). Differences between groups were examined for statistical significance using one‐way ANOVA with Tukey’s *post hoc* test.

## Results

### Aaptamine inhibited the activities of AChE and BuChE

**Figure 2.**
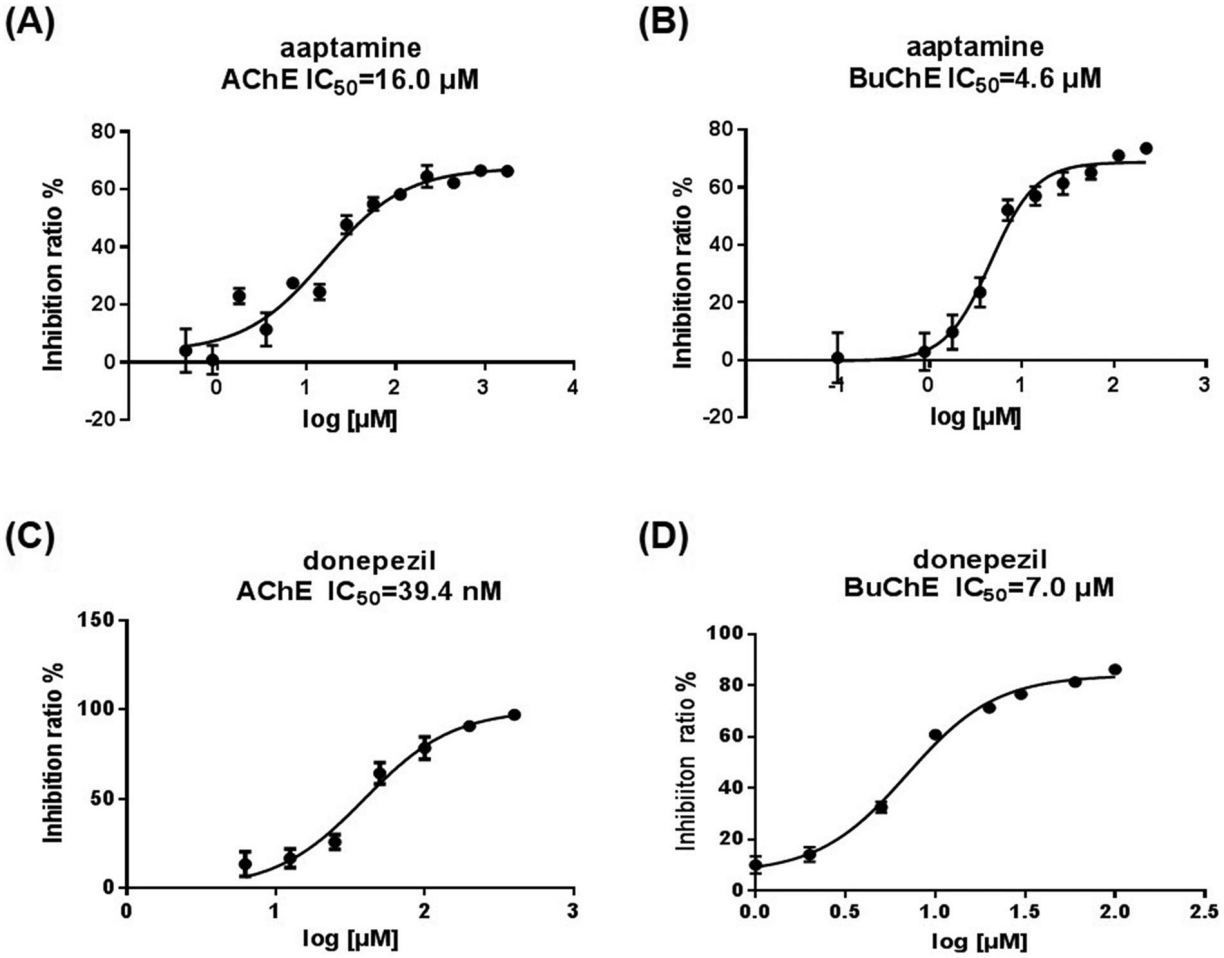
Dose-response and IC_50_ values of aaptamine and donepezil against AChE and BuChE.

As shown in Figure 2, aaptamine showed inhibitory activities towards both AChE and BuChE, with IC_50_ values of 16.0 and 4.6 μM, respectively. Donepezil strongly inhibited AChE, with an IC_50_ value of 39.4 nM, and moderately inhibited BuChE, with an IC_50_ value of 7.0 μM. Interestingly, aaptamine exhibited a more potent inhibitory effect on BuChE than on AChE.

### Aaptamine interacted with the key sites of AChE and BuChE

To validate the experimental results and determine plausible binding interactions of aaptamine with AChE and BuChE, molecular docking studies were performed.

To validate whether the docking software was reliable for AChE (PDB code: 4EY7) and BuChE (PDB code: 4TPK), the crystal pose of donepezil was initially re-docked into the binding sites of AChE; similarly, *N*-[(1-(2,3-dihydro-1H-inden-2-yl)piperidin-3-yl)methyl]-*N*-(2-methoxyethyl)-2-naphthamide was re-docked into the pocket of BuChE using surflex-docking. Then, the redocked conformations were compared with the original crystallographic conformations of the ligands. The re-docked compounds and crystal ligands in the complex were almost completely superimposable, thereby indicating that the surflex-docking method and parameters used were reasonable and reliable.

**Figure 3.**
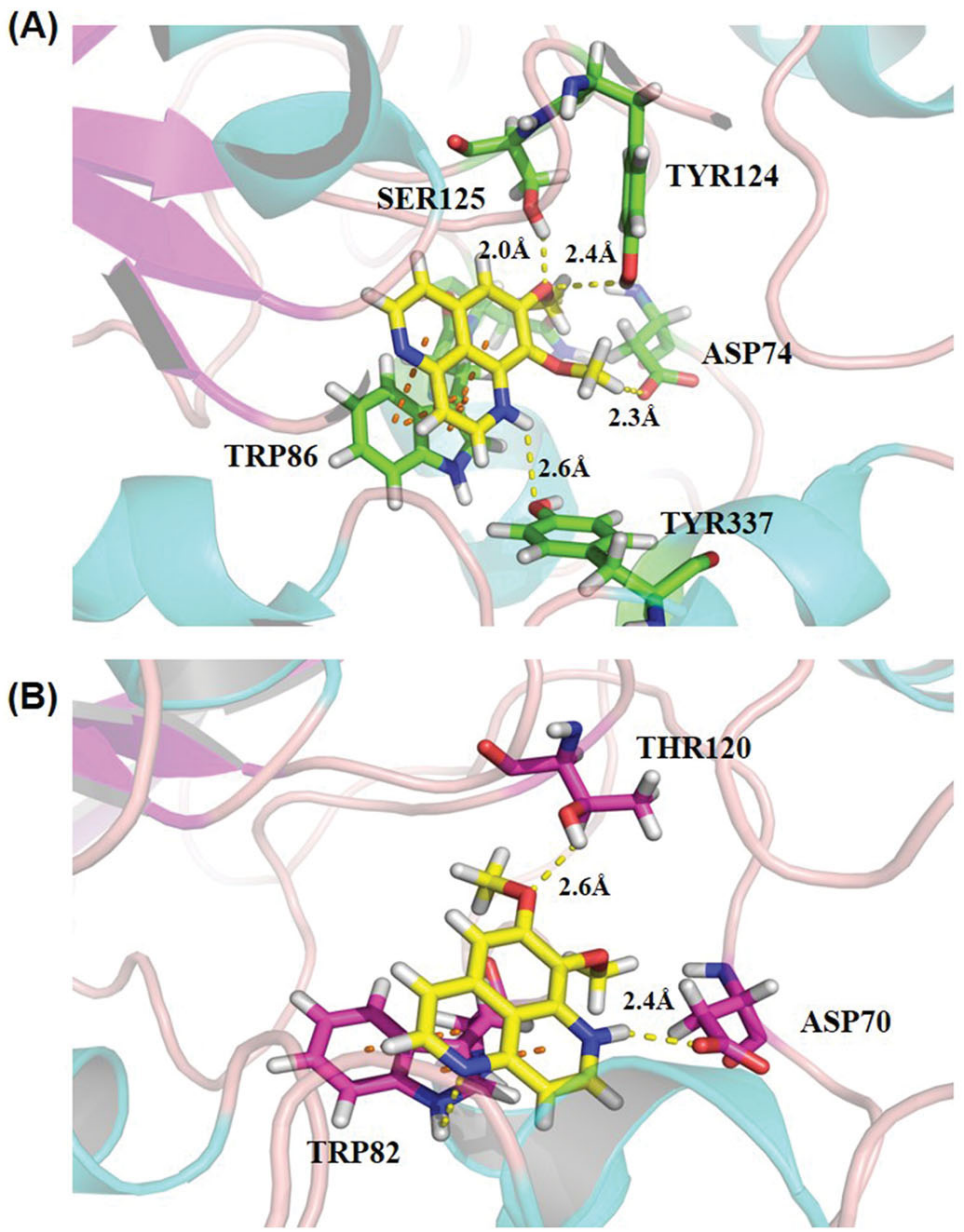
(A) The binding model between aaptamine and AChE. (B) Binding model between aaptamine and BuChE. Orange dashed line: π–π conjugate effect; Yellow dashed line: hydrogen bond. Yellow sticks structure: compound aaptamine; Green sticks structure: protein AChE; Magenta sticks structure: protein BuChE.

As shown in Figure 3(A), the nuclear ring of aaptamine formed a π-π conjugation with TRP86 in AChE, thus stablizing the nuclear ring conformation. In addition, the –NH functional group of aaptamine forms one hydrogen bond (2.9 Å) with TYR337, an important amino acid in the active site (Cheung et al. 2012). Moreover, the methoxy substituents provided polar interactions with peripheral anionic site (PAS) residues, namely TYR124 and SER125, by forming stable hydrogen bonds at distances of 3.1 and 2.2 Å with a bond angle of 75.3° (Demir Özkay et al. 2016). Therefore, aaptamine could bind simultaneously to both the catalytic active sites (CAS) and PAS of AChE. The docking scores of aaptamine and donepezil with AChE were 6.0 and 9.3, respectively. As shown in Figure 3(B), the compound was embedded in the cavity of BuChE, thereby forming both π–π conjugation and hydrogen bonding with TRP82, a crucial anionic site of PAS that plays an important role in controlling the opening and closing of CAS (Delogu et al. 2016). On the other side, aaptamine developed two strong hydrogen bonds at the same distance of 2.8 Å with ASP70 and THR120. These valuable interactions might explain the strong inhibitory effect of aaptamine on BuChE (Kim et al. 2016; Pang et al. 2017). Furthermore, the docking score of aaptamine with BuChE was 7.9 as compared to 9.4 of *N*-[(1-(2,3-dihydro-1H-inden-2-yl)piperidin-3-yl)methyl]-*N*-(2-methoxyethyl)-2-naphthamide, which is a nanomolar-selective BuChE inhibitor.

### Aaptamine inhibited AChE and BuChE with mixed-type kinetics

**Figure 4.**
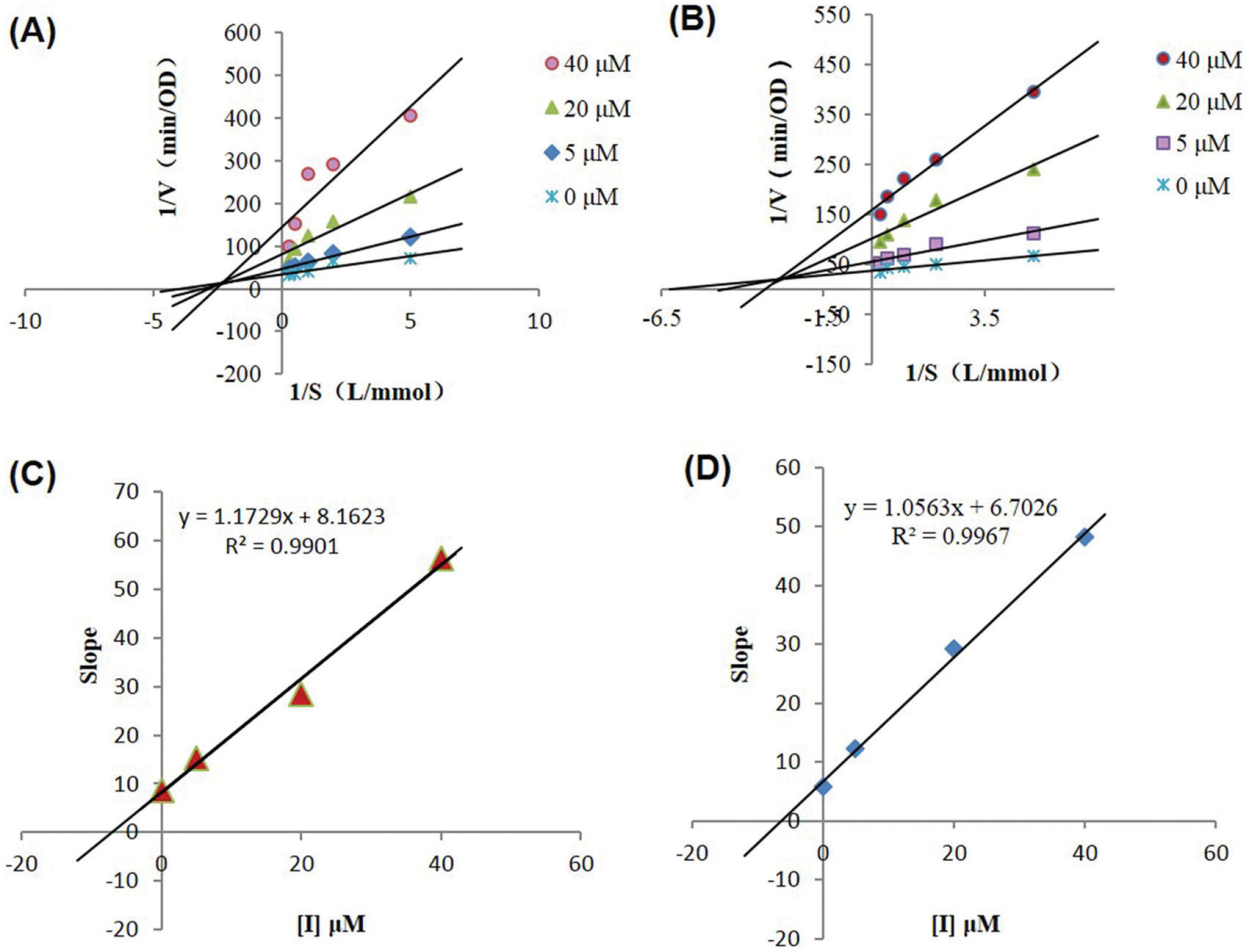
(A) Lineweaver–Burk plot for the inhibition of AChE by aaptamine at different concentrations for substrate ATChI. (B) Secondary plot for calculation of the steady-state inhibition constant (*Ki* = 1.74 ± 0.04 μM against AChE) of aaptamine. (C) Lineweaver–Burk plot for the inhibition of BuChE by aaptamine at different concentrations for substrate BTChI. (D) Secondary plot for calculation of the steady-state inhibition constant (*Ki* = 1.59 ± 0.02 μM against BuChE) of aaptamine.

The inhibitory effects of aaptamine on AChE and BuChE were investigated using Lineweaver–Burk plots. As shown in Figure 4, the four plots were linear and intersected in the second quadrant of the coordinate axis, which characterizes a typical mixed inhibitory type (Figure 4(A,B)). In addition, the Lineweaver–Burk plot showed increased slopes (decreased *V*_max_) and intercepts (higher *K_m_*) at higher concentrations of aaptamine, thus indicating a mixed-type inhibition (Sağlık et al. 2016). If the molecule has both binding sites on CAS and PAS, then enzymatic inhibition may be determined as a type of mixed inhibition (Hussein et al. 2018). Accordingly, the present enzyme kinetics study suggested that aaptamine interacted with both the functional sites of CAS and PAS of AChE and BuChE. The PAS sites also improved the catalytic efficiency, thus leading to higher inhibitory effects (Pang et al. 2017).

The steady-state inhibition constant, *K_i,_* can be used to effectively measure the affinity of the enzyme and its substrate. Secondary plots of the slopes of Lineweaver–Burk plots against inhibitor concentration showed aaptamine *K_i_* values of 6.96 ± 0.04 μM for the inhibition of AChE and 6.35 ± 0.02 μM for BuChE (Figure 4(C,D)); thus, aaptamine is revealed to firmly bind to both AChE and BuChE.

### Aaptamine directly interacted with AChE and BuChE

**Figure 5.**
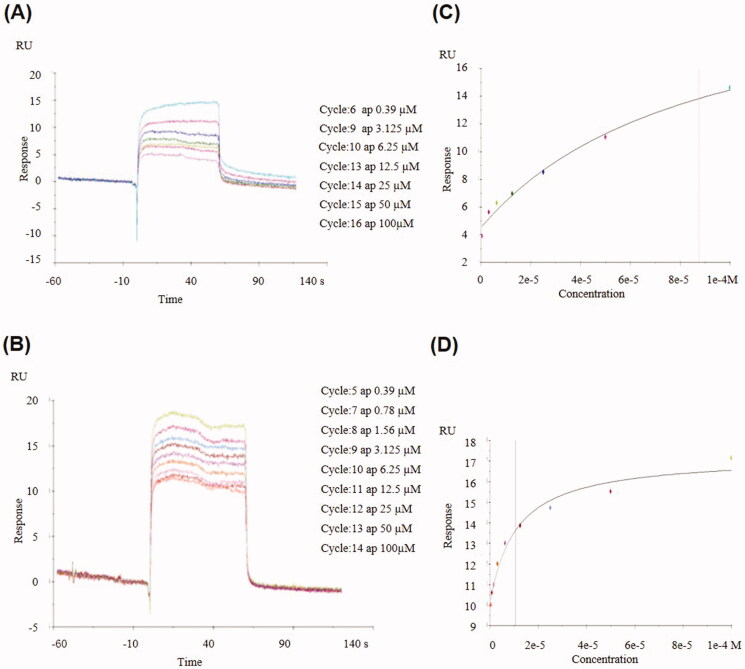
(A) Biacore analysis of aaptamine binding to AChE. (B) Steady state of affinity fitting of aaptamine – AChE interaction. (C) Biacore analysis of aaptamine binding to BuChE. (D) Steady state of affinity fitting of aaptamine – BuChE interaction.

To further validate the direct interaction of aaptamine with AChE and BuChE, we used AChE and BuChE proteins to perform SPR using Biacore T100. The kinetics of the binding reaction was determined by injecting different concentrations of aaptamine over pure AChE or BuChE immobilized on the chip surface. The interaction curves (Figure 5(A,B)) and steady-state affinity fitting (Figure 5(C,D)) indicated that aaptamine was directly bound to AChE and BuChE in a concentration-dependent manner with micromolar binding affinity; the *K_d_* (equilibrium dissociation constant) values were 87.6 and 10.7 μM, respectively.

### Aaptamine showed dyskinesia rehabilitation effects in zebrafish

**Figure 6.**
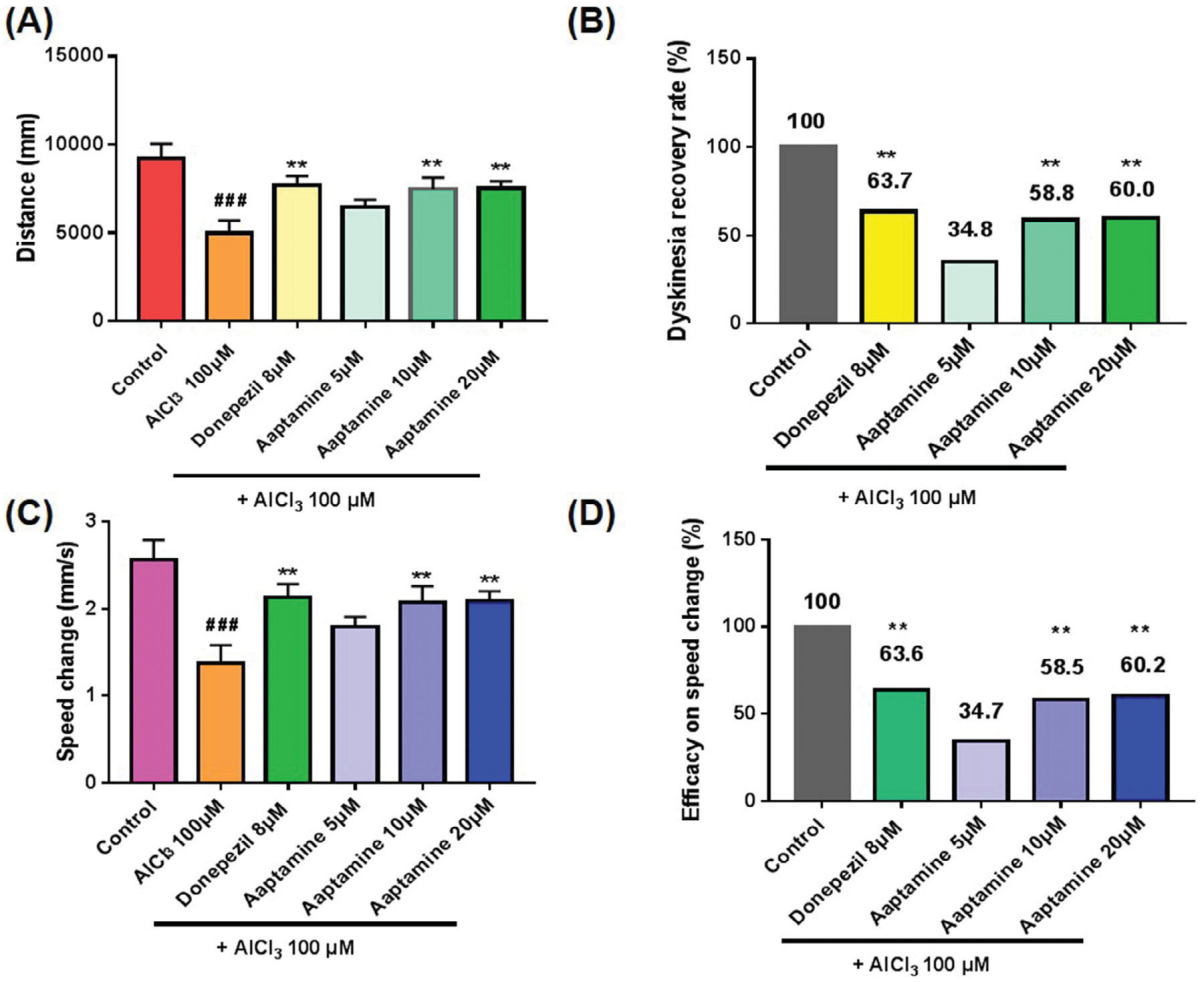
(A,B) The effect of aaptamine on motility distance of AD zebrafish ^###^*p* < 0.001 *vs.* Control group, **p < 0.01 *vs.* AlCl_3_ group. (C,D) Effect of aaptamine on speed change in AD zebrafish. ^###^*p* < 0.001 *vs.* Control group, **p < 0.01 *vs*. AlCl_3_ group.

Zebrafish models have attracted considerable attention and have been widely used for the rapid screening of potentially therapeutic anti-AD agents in recent years. To further evaluate the anti-AD effect of aaptamine, an AlCl_3_-induced zebrafish AD model was established to study the dyskinesia and reaction recovery capacity before treatment with 5, 10 and 20 μM aaptamine. Furthermore, 8 μM donepezil, a well-recognized drug for treating AD, was used as the positive control. This experiment was based on the behaviour of the zebrafish; their swimming distance within a certain period of time was considered as an indicator, that is, the greater the distance, the better the ability of aaptamine to ameliorate dementia in the model (Sang et al. 2019). As shown in Figure 6(A), the distance of AlCl_3_-induced AD zebrafish (model group, 4968 mm) was significantly shorter than that of untreated zebrafish (control group, 9219 mm; *p* < 0.001). This indicated the successful establishment of an AlCl_3_-induced AD model in zebrafish. The distance of the 8 μM donepezil treatment group increased to 7675 mm (*p* < 0.01) while the dyskinesia recovery rate was 63.7% as compared with that of the model group, thereby implying that donepezil could improve the exercise capacity of the zebrafish AD model. When treated with 5, 10 and 20 μM aaptamine, the distances of the three treatment groups were 6447 mm (*p* > 0.05), 7466 mm (*p* < 0.01) and 7517 mm (*p* < 0.01), and their dyskinesia recovery rates were 34.8, 58.8 and 60.0% (Figure 6(A,B)), respectively. These data indicate that the therapeutic effects of 10 and 20 μM aaptamine were similar to those of donepezil. Accordingly, aaptamine improved the recovery rate of dyskinesia in zebrafish with AlCl_3_-induced AD.

To evaluate whether aaptamine could improve the response efficiency of zebrafish with AD, a series of experiments were conducted. As shown in Figure 6(C), the speed change of the AlCl_3_-induced AD zebrafish model (model group, 1.38 mm/s, *p* < 0.001), which was stimulated by light, was significantly shorter than that of untreated zebrafish (control group, 2.56 mm/s), thus indicating that the zebrafish AD model responded efficiently. After treatment with 8.0 μM donepezil, the speed change in AD zebrafish increased to 2.13 mm/s (donepezil group, *p* < 0.01), and the efficacy was 63.6%, thus indicating that donepezil also improved the response efficiency of AlCl_3_-induced AD zebrafish. When treated with 5, 10 and 20 μM aaptamine, the speed change of AD zebrafish increased to 1.79 (*p* > 0.05), 2.07 (*p* < 0.01) and 2.09 mm/s (*p* < 0.01) (Figure 6(C)), and the efficacies were 34.7, 58.5 and 60.2% (Figure 6(D)), respectively. Among these groups, the medium and high doses of aaptamine (10 and 20 μM) showed a similar therapeutic effect in terms of improving response efficiency as compared with the donepezil group.

## Discussion

In this study, we identified new activities of aaptamine as a valid inhibitor of AChE and BuChE, as well as a potential therapeutic candidate for the treatment of AD. Aaptamine, a typical marine alkaloid, is the main constituent of the marine sponge *Aaptos*. Previously, aaptamine was well-known as a potent antitumor agent *via* multiple mechanisms, such as activating the tumour suppressor p21, thus inducing cell cycle arrest and cell death as well as inhibiting the PI3K/AKT/GSK3β signalling pathway (Aoki et al. 2006; Tsukamoto et al. 2010; Jin et al. 2011; Stuhldreier et al. 2015). Many studies have revealed that AChE and BuChE are key players involved in the pathology of AD and may serve as potential therapeutic targets (Hampel et al. 2018). Currently, only three cholinesterase inhibitors approved by the FDA, namely donepezil, rivastigmine and galantamine are in clinical use. Among them, donepezil and galantamine selectively inhibited AChE (IC_50_ values of 0.022 and 0.8 μM), whereas rivastigmine non-selectively inhibited both AChE and BuChE (IC_50_ values of 4.15 and 0.037 μM) (Moodie et al. 2019). BuChE has been reported to compensate for the role of AChE in hydrolysing ACh during the middle and late stages of AD (Zhou and Huang 2022). Therefore, the appropriate dual inhibition of AChE and BuChE is an ideal treatment strategy for AD. However, rivastigmine is the only dual AChE-BuChE inhibitor that has been approved by the FDA for treating all stages of AD (Kandiah et al. 2017). Although new cholinesterase inhibitors, including naturally occurring or synthesized compounds, have been reported, of which only a small number are dual AChE-BuChE inhibitors (Anand and Singh 2013; Li et al. 2017; Moodie et al. 2019). In this study, we found that aaptamine had inhibitory activities towards both AChE and BuChE, with IC_50_ values of 16.0 and 4.6 μM, respectively. This indicated the promising prospect of aaptamine in AD treatment which may provide a new scaffold for the development of dual-cholinesterase inhibitors.

The overall structures of AChE and BuChE are very similar; they share almost 65% of their homologous amino acid sequences (Pang et al. 2017). Both contain a CAS, a deep gorge, and a PAS. The CAS at the bottom of the channel was the binding site for the substrates. PAS can facilitate the movement of ACh towards CAS and therefore contribute to improving the catalytic efficiency of ACh (Guo et al. 2020). Molecular docking showed that aaptamine can interact with key amino acid residues in the CAS and PAS sites of AChE and BuChE through hydrogen bonds or π–π stacking interactions. In addition, the findings were in good agreement with kinetic studies with mixed-type inhibition of the two cholinesterases, whereby aaptamine was also verified to interact with CAS and PAS simultaneously. SPR assay indicated that aaptamine was bound to BuChE with a lower *K_d_* value, which provides an explanation for the higher inhibitory effect of aaptamine towards BuChE than AChE. Furthermore, AChE inhibitors have been reported to usually possess a basic centre that interacts with CAS, a core ring system that binds to PAS, and a linker between the core ring system and the basic centre to fulfil structural requirements. For example, donepezil contains an indanone core ring, methylene linkers, and a basic benzylpiperidine centre. The benzylpiperidine moiety of donepezil binds to CAS, whereas the indanone moiety interacts with PAS (Hussein et al. 2018). In addition, the bulky moieties were reported to possess good BuChE inhibitory activity, which may be related to BuChE having a more spacious cavity to optimally accommodate compounds with larger substituents (Krátký et al. 2016). In contrast, the aaptamine structure is simpler and possesses several sites for improvement. In further research, the structure of aaptamine can be optimized based on empirical knowledge to add more groups to the mother nucleus or change the methoxy group to other diverse side chains to obtain more potent AChE and BuChE dual-target inhibitors.

In addition to enhancing the catalytic efficiency of ACh, the PAS of AChE was also found to induce the formation of A*β* protein and accelerate its precipitation (Pietsch and Gütschow 2005). Thus, AChE inhibitors with dual binding sites will not only stimulate the cholinergic system to alleviate cognitive deficits in AD patients, but also inhibit the production or aggregation of A*β* (Castro and Martinez 2006). Recent studies have indicated that AChE inhibitors could also reduce the aggregation of tau proteins in the regions of the entorhinal cortex and hippocampus in amyloid-positive participants, which may exhibit some neuroprotective effects in patients within the AD continuum (Yasuno and Minami 2021). Therefore, aaptamine is a promising compound for the development of new anti-AD drugs. Nevertheless, the capacity of aaptamine to inhibit A*β* peptide fibrillogenesis and tau protein aggregation should be further examined through *in vitro* and *in vivo* experiments.

Zebrafish are rapidly emerging as valuable models for AD research (Pitchai et al. 2019). A search for available scientific literature in the PubMed database using the term, ‘zebrafish + Alzheimer’s disease’, revealed 356 publications, with 288 being original research articles (12 April 2022). AlCl_3_ induced AD-like behaviour in locomotor activity tests in zebrafish. Accordingly, swimming distance and speed change are the two main parameters that reflect model validity and drug effects. To further evaluate the anti-AD effects of aaptamine, the AlCl_3_-induced AD zebrafish model was used to conduct *in vivo* experiments. We chose an 8 μM dose of donepezil as the positive control according to the literature (Pan et al. 2019; Sang et al. 2019, 2020). The results showed that AlCl_3_-induced zebrafish AD model group was deficient in swimming distance and speed changes as compared to the control group. Furthermore, zebrafish showed significant dyskinesia and response recovery after treatment with donepezil (8 μM) or aaptamine (10 and 20 μM), thus indicating that 10 and 20 μM aaptamine possesses a therapeutic effect similar to that of donepezil. Aaptamine was also reported to have a weak inhibitory effect on nicotinic acetylcholine receptors (nAChRs) of *Torpedo californica* Ayres (Torpedinidae) and humans and would cause some peripheral effects, such as muscle diastole, which seems contradictory to the results of the assays in this study (Kudryavtsev et al. 2014). This further implied that aaptamine improved AD-associated dyskinesia, probably by inhibiting the activities of cholinesterase. Although zebrafish is a very promising AD model, especially as a formidable high-throughput drug screening tool, there are still some limitations. For example, whether zebrafish can be employed to model late-onset AD is debateable because they have the ability to regenerate neurons at a faster rate than that in mammals (Newman et al. 2014). Therefore, in depth *in vivo* studies of aaptamine and its derivatives in mouse AD models should be undertaken in the future.

## Conclusions

In this study, we focussed on the dual inhibition effects of aaptamine on AChE and BuChE. Aaptamine displayed potential mixed-type inhibitory activities towards AChE and BuChE by simultaneously binding with the PAS and CAS sites of the two cholinesterases. Importantly, the *in vivo* experiments suggested that aaptamine could significantly improve dyskinesia recovery and response efficiency in the AlCl_3_-induced zebrafish AD model. To the best of our knowledge, aaptamine represents a novel class of potential AChE/BuChE dual-targeted inhibitors for AD treatment. These data may pave the way for the researchers to optimize the structure of aaptamine to enhance its pharmacological effect.
